# Food animals as reservoirs and potential sources of multidrug-resistant diarrheagenic *E. coli* pathotypes: Focus on intensive pig farming in South Africa

**DOI:** 10.4102/ojvr.v89i1.1963

**Published:** 2022-01-20

**Authors:** Shima E. Abdalla, Akebe L.K. Abia, Daniel G. Amoako, Keith Perrett, Linda A. Bester, Sabiha Y. Essack

**Affiliations:** 1 Antimicrobial Research Unit, College of Health Sciences, University of KwaZulu-Natal, Durban, South Africa; 2 Epidemiology Section, KwaZulu-Natal Agriculture and Rural Development-Veterinary Service, Pietermaritzburg, South Africa; 3 Biomedical Resource Unit, College of Health Sciences, University of KwaZulu-Natal, Durban, South Africa

**Keywords:** diarrheagenic *E. coli*, multidrug resistance, intensive pig farming, farm-to-fork, low-income countries, South Africa, multiple-antibiotic resistance index, biosecurity

## Abstract

**Background:**

Diarrheagenic *E. coli* (DEC) strains are a major cause of diarrheal diseases in both developed and developing countries. Healthy asymptomatic animals may be reservoirs of zoonotic DEC, which may enter the food chain via the weak points in hygiene practices.

**Aim:**

We investigated the prevalence of DEC along the pig production continuum from farm-to-fork.

**Methods:**

A total of 417 samples were collected from specific points along the pig production system, that is, farm, transport, abattoir and food. *E. coli* was isolated and enumerated using Colilert. Ten isolates from each Quanti-tray were selected randomly and phenotypically identified using eosin methylene blue agar selective media. Real-time polymerase chain reaction (PCR) was used to confirm the species and to classify them into the various diarrheagenic pathotypes. Antimicrobial susceptibility was determined against a panel of 20 antibiotics using the Kirby-Bauer disk diffusion method and EUCAST guideline.

**Results:**

The final sample size consisted of 1044 isolates, of which 45.40% (474/1044) were DEC and 73% (762/1044) were multidrug-resistant. Enteroinvasive *E. coli* (EIEC) was the most predominant DEC at all the sampling sites.

**Conclusion:**

The presence of DEC in food animal production environments and food of animal origin could serve as reservoirs for transmitting these bacteria to humans, especially in occupationally exposed workers and via food. Adherence to good hygienic practices along the pig production continuum is essential for mitigating the risk of transmission and infection, and ensuring food safety.

## Introduction

Intensive pig production can be defined as raising a large number of animals on limited land (Mennerat et al. 2010) to increase profits and ensure sustainability in meat production (Noya et al. 2017). In some countries, intensive pig farms are further integrated with meat-processing industries, forming supply chains (Davies 2011). Additionally, intensive pig production is practised under strict conditions such as temperature control, reduction of contact between animals and waste, improvement of effluent treatment, parturition control with human intervention and the use of vaccines. However, these measures are inadequate to achieve ideal sanitary conditions (Alustiza et al. 2012), and livestock still act as intermediary or amplifier hosts of pathogens transmitted to humans (Jones et al. 2013). To prevent the introduction and spread of infectious diseases in pig production, biosecure environments and strict hygiene conditions have been adopted (Julio Pinto & Santiago Urcelay 2003). Biosecurity prevents direct and indirect disease transmission between animals from the same and between different batches or farms (Sahlstrom et al. 2014). Within meat processing plants, pathogens can easily be transferred to meat from the animals’ gastrointestinal tract, environment and meat handlers’ hands, especially under poor sanitary conditions (Ncoko, Jaja & Oguttu 2020).

Although most *Eschericheia coli* (*E. coli*) strains are commensals and live harmlessly in the colon of humans and other animals, several pathogenic *E. coli* strains cause intestinal and extraintestinal diseases in healthy and immunocompromised humans (Gomes et al. 2016) and animals. These pathogenic *E. coli* strains carry several different virulence factors, controlled by genes located on chromosomes, plasmids or phages (Borges et al. 2012). Pathogenic *E. coli* can cause different dieases and affect both gastriontestinal and extraintestinal sites. Several gastointestinal *E. coli* pathotypes contribute to diarrhea (Croxen et al. 2013).

Diarrheagenic *E. coli* (DEC) strains are considered major causes of diarrheal diseases in developed and developing countries (Aijuka et al. 2018; Estrada-Garcia & Navarro-Garcia 2012). Foodborne diseases resulting from the consumption of food contaminated by DEC have been recognised amongst the most challenging health issues worldwide (Galli et al. 2016).

Members of the DEC group are classified into six pathotypes, that is, enteroaggregative *E. coli* (EAEC), enteropathogenic *E. coli* (EPEC), enterotoxigenic *E. coli* (ETEC), enteroinvasive *E. coli* (EIEC), diffusely aggregative *E. coli* (DAEC) and enterohemorrhagic/Shiga toxin-producing *E. coli* (EHIEC/ STEC) (Acosta et al. 2016). The EPECis associated with infantile and persistent diarrhea. It is mainly detected by the presence of the eae gene (Ochoa & Contreras 2011). The ETEC is an important cause of diarrhea in children and travellers (Isidean et al. 2011). It is characterised by the presence of a heat-stable (*ST*) and/or heat-labile (*LT*) enterotoxin gene, encoding colonisation factors and toxin production (Gomes et al. 2016). This pathotype remains a problem for humans, pigs and calves. It causes diarrhea in neonatal and recently weaned piglets, which is considered one of the most important diseases affecting pig farming economically (Melkebeek, Goddeeris & Cox 2013). The EIEC causes dysentery in humans, is closely related to Shigella (Hosseini Nave et al. 2016), and can be distinguished from other *E. coli* by the detection of the ipaH gene (Van den Beld & Reubsaet 2012). The EAEC has been associated with persistent diarrhea in children, travellers and humans with immunodeficiency virus infections; it induces chronic inflammation in the absence of dysentery (Okhuysen & Dupont 2010). The EAEC is characterised by the transcriptional activator encoding aggR gene (Wang et al. 2017). The EHEC/STEC infections range from mild to severe, complicated, bloody diarrhea and haemolytic uremic syndrome (Friesema et al. 2011). This pathotype produces one or more types of Shiga toxin (Stx1 and/or Stx2) (Dias et al. 2016). The DAEC pathotypes consist of a heterogeneous group of organisms with variable virulence factors that may play a role in causing sporadic diarrheal illnesses, particularly in paediatric patients (Wang et al. 2017), and are detected by the presence of the *daaD* gene (Riveros et al. 2017).

Apart from causing infections, *E. coli* pathotypes have been reported globally for their resistance to numerous antibiotics, including those used in clinical medicine. The escalation of antibiotic resistance in pathogenic bacteria is a major global public health concern (Fernandez, Bert & Nicolas-Chanoine 2016) because it has led to prolonged illness and higher treatment failure rates (Van den Honert, Gouws & Hoffman 2018). This concern is further exacerbated by the emergence of multidrug-resistant (MDR) bacteria, resulting in reduced treatment options for the infections they cause (Lammie & Hughes 2016). The MDR *E. coli* has been increasingly isolated from livestock and animal products (Kallau et al. 2018). This increase has been attributed to increased antibiotic use and sub-optimal biosecurity programmes on farms (Mbelle et al. 2019).

Human exposure to MDR DEC could lead to disease outbreaks with severe adverse public health consequences. Knowledge of the prevalence and distribution of these MDR pathotypes within the pig production continuum is therefore imperative. We investigated the prevalence of MDR DEC pathotypes in a pig production continuum using a farm-to-fork approach. Such information could help identify areas needing attention across the continuum, to prevent the spread of these infectious agents to humans through both meat products and environmental exposure.

## Materials and methods

### Study design, sample collection and enumeration of *E. coli*

The study was a longitudinal study conducted over 18 weeks (September 2018 – January 2019) covering the farm-to-fork pig production continuum. It included sampling on the farm, transport system (truck) and attached abattoir, using the guidelines of the World Health Organization Advisory Group on Integrated Surveillance of Antimicrobial Resistance (WHO-AGISAR) (WHO 2017).

A total of 417 samples were collected and processed as previously described (Abdalla et al. 2021). Samples consisted of faeces, litter and slurry (farm), swabs of transport vehicles, and ceacal, carcass swabs and final meat cut swabs (abattoir). Briefly, the defined substrate ColilertTM-18 system from IDEXX (IDEXX Laboratories (Pty) Ltd., Johannesburg, South Africa) was used to detect and quantify *E. coli* according to manufacturer instructions. All processed samples were incubated for 18–24 h at 37 °C and examined under UV light for fluorescence.

### *E. coli* confirmation and detection of pathotypes

Pure *E. coli* strains were obtained from fluorescent Quanti-Tray wells as previously described (Abia, Ubomba-Jaswa & Momba 2015). Ten isolates were randomly selected from each of the 417 samples and phenotypically identified on eosin methylene blue agar (HiMedia Laboratories Pvt. Ltd., Mumbai, India). The isolates were further streaked on nutrient agar (Neogen, Lansing, Michigan, United States) and incubated at 37 °C for 24 h. Deoxyribonucleic acid (DNA) was extracted from the isolates using the boiling method (Amoako et al. 2019). Real-time polymerase chain reactions were used to confirm *E. coli* and determine the various DEC pathotypes targeting specific genes (Appendix 1). The reaction mixtures and thermal cycling conditions were as previously described (Abia et al. 2015), except for the master mix where the Luna® Universal qPCR Master Mix (New England Biolabs, Ipswich, Massachusetts, United States) was used in this study. After the final extension step, a melt curve was generated and analysed as previously described (Molechan et al. 2019). All reactions were performed on a QuantStudioTM 5 (ThermoFischer Scientific, Bedford, MA, USA). The DNA from reference *E. coli* strains was used as positive controls (Table 1-A1), whilst the reaction mixture with no DNA (replaced with nuclease-free water) was used as a no template control. All controls were obtained in-house from the Antimicrobial Research Unit microbial bank.

### Antibiotic susceptibility testing

The antibiotic susceptibility profiles of the confirmed diarrheagenic isolates were determined against 20 antibiotics using the disk diffusion method on Muller Hinton Agar (Oxoid, Basingstoke, Hampshire, England) as previously described (Abdalla et al. 2021). The *E. coli* ATCC® 25922 was used for quality control. The diameters of the zones of inhibition were measured and interpreted according to the European Committee on Antimicrobial Susceptibility testing breakpoints (EUCAST 2017). The Clinical and Laboratory Standards Institute guidelines (CLSI) were used (CLSI 2017) for antibiotics that did not have published breakpoints in the EUCAST guidelines. All the antibiotic discs were purchased from Oxoid (Basingstoke, Hampshire, England). Isolates showing resistance to ≥ 1 agent in > 3 distinct antibiotic classes were considered as MDR (Amoako et al. 2016). A multiple-antibiotic resistance index (MARI) was calculated for these isolates to ascertain whether these isolates originated from high antibiotic use environments (Abdalla et al. 2021).

### Statistical analysis

The data were analysed using the Statistical Package for the Social Science (SPSS) version 26 (IBM Corporation, Armonk, New York, United States). Descriptive statistics were used to describe the frequency of DEC along the pork production chain. The statistical significance of the differences in counts and DEC prevalence between different sources was determined using the one-way analysis of variance (ANOVA) with Tukey’s Honestly Significant Difference (HSD) post-hoc test. A *p*-value < 0.05 was considered statistically significant.

### Ethical considerations

Ethical approval was obtained from the Animal Research Ethics Committee (AREC 073/016PD) and the Biomedical Research Ethics Committee (BCA444/16) of the University of KwaZulu-Natal. A Section 20A permit (12/11/1/5) was further obtained from the South African National Department of Agriculture, Forestry and Fisheries.

## Results

### Mean *E. coli* count per sampling site

**FIGURE 1:**
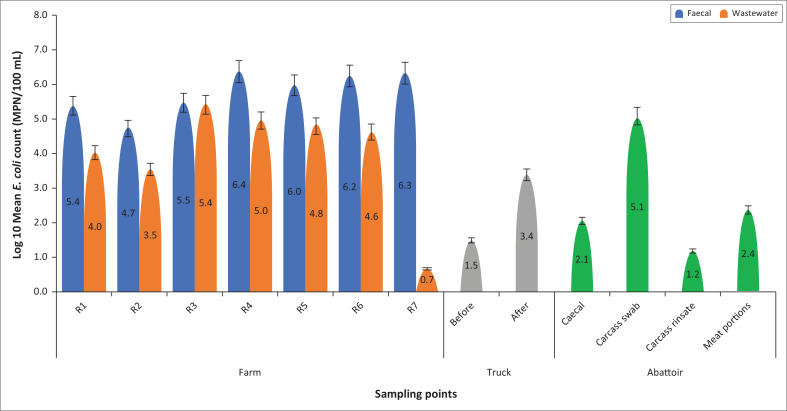
*E. coli* counts along the pig production continuum Farm (Round 1–7), Truck, Abattoir. Error bars represent 5% error.

The *E. coli* was isolated from all the samples collected in this study. The mean *E. coli* concentrations per sampling site are shown in Figure 1. The highest mean *E. coli* count was recorded in the faecal samples (1.59 × 10^6^ MPN/100 mL), whilst the lowest was recorded in truck samples. There was an overall statistically significant difference (*p* = 0.002) between the *E. coli* counts from the different sampling points (Table 2-A1). The post-hoc analysis revealed a statistically significant difference in the overall *E. coli* count between the farm and truck (*p* = 0.045), and the farm and abattoir (*p* = 0.004). However, no statistically significant difference was observed between the truck and abattoir *E. coli* counts (*p* = 0.183).

### Identification of *E. coli* pathotypes

A total of 1044 isolates were confirmed as *E. coli* using real-time PCR. Pathotyping using pathotype-specific genes showed that 45.40% (474) of the isolates carried at least one of the virulence genes tested (Table 1). The highest percentage of DEC was isolated on the farm, whilst the lowest was found at the transport (truck). The EIEC at 19% was the most prevalent pathotype, whilst EHEC was the least prevalent (0.1%).

**TABLE 1:** Distribution of diarrheagenic *E. coli* pathotypes along the farm-to-fork continuum.

Pathotype	Target gene	Sampling point			Total	
		Farm	Truck	Abattoir	Number	*%*
EPEC/EHEC	*Eae*	154	-	1	155	14.7
ETEC	*it-st*	44	-	-	44	4.2
EAEC	*eagg*	9	-	-	9	0.9
DAEC	*daaE*	36	-	-	36	3.4
EIEC	*ipah*	132	7	62	201	19.0
EHEC	*stx/ flicH7*	37	-	-	37	3.5

EPEC, enteropathogenic *E. coli*; EHEC, enterohemorrhagic *E. coli*; ETEC, enterotoxigenic *E. coli*; EAEC, enteroaggregative *E. coli*; DAEC, diffusely aggregative *E. coli*; EIEC, enteroinvasive *E. coli.*

Like with the abundance of *E. coli* across the continuum, there was an overall statistically significant difference (*p* = 0.000) between the prevalence of the DEC pathotypes from the different sampling points (Table 3-A1). Similarly, Tukey’s HSD post-hoc analysis of the DEC pathotypes prevalence was statistically significantly different between the farm and truck (*p* = 0.000), farm and abattoir (*p* = 0.039) and truck and abattoir (*p* = 0.006) (Table-3A1).

### Antibiotic susceptibility profiles

Only 1% (5 isolates) of the total DEC was susceptible to all the antibiotics been tested.

**FIGURE 2:**
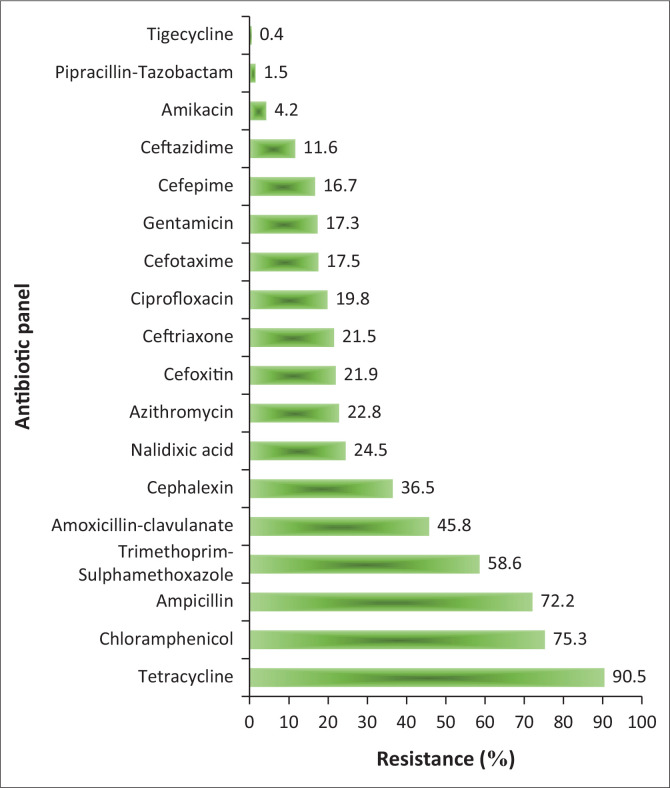
Overall antibiotic resistance in diarrheagenic *E. coli* pathotypes across the pig production chain.

**FIGURE 3:**
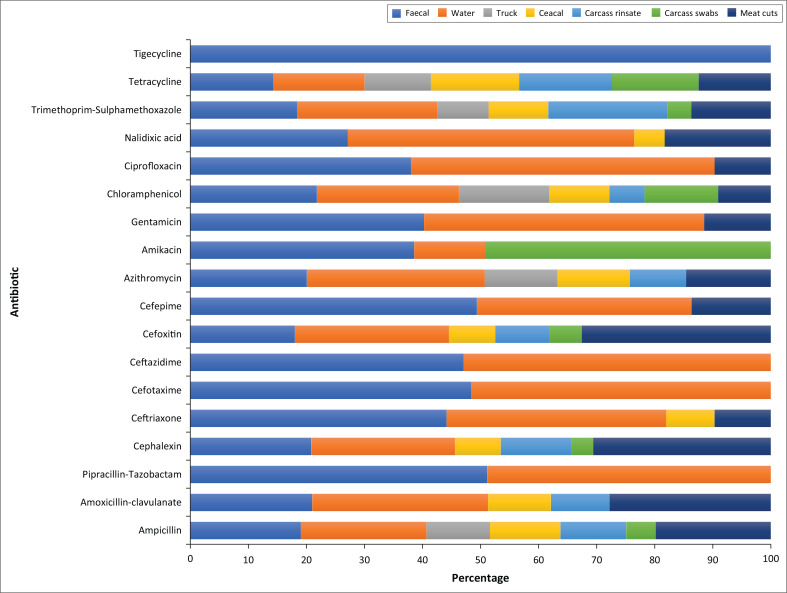
Percentage resistance to each antibiotic differed by source.

Overall, the highest resistance was against tetracycline 90.5% (429), whilst all the isolates were susceptible to meropenem (Figure 2); the percentage resistance to each antibiotic differed by source (Figure 3).

**FIGURE 4:**
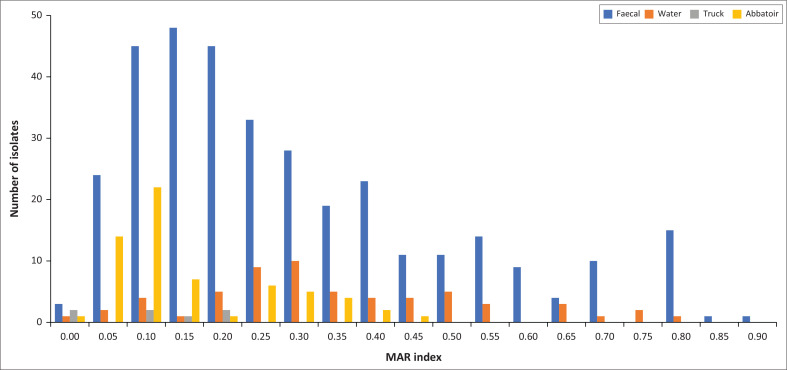
The multiple antibiotic resistance (MAR) indices of *E. coli* sampled across different sources (farm, water, truck and abattoir).

The MDR was detected in 73.84% (350/474) of the total isolates, most of which were from the farm (92.29%; 323/350). Most of these isolates (69.71%; 244/350) recorded a MARI above 0.2 (Figure 4). The highest MARI was 0.9 (resistance to 18/20 antibiotics tested), recorded by an EHEC strain on the farm (Table 4-A1).

## Discussion

This study investigated the prevalence of DEC pathotypes and their antibiotic resistance profiles in intensive pig farming in uMgungundlovu District, KwaZulu-Natal, South Africa, using the farm-to-fork approach. The *E. coli* was recovered from all the samples along the pig production continuum. Of the total number of 1044 *E. coli* isolates constituting the sample size across the continuum, 45% were DEC, with EIEC being the most prevalent pathotype. Also, 99% of the isolates were resistant to at least one of the antibiotics tested, 73.84% were MDR, most recording a MARI greater than 0.2.

### Enumeration of *E. coli* across the continuum

The enumertion results reported in this study are lower than those previously reported in an earlier study in South Africa (King & Schmidt 2017) and Bulgaria (Petkov et al. 2006). However, the reported results in the current study were higher than those reported in a study involving 13 pigs farms in Australia (Chinivasagam et al. 2004). Despite the discrepancies between our study and the previous study, the *E. coli* abundance in the faeces observed in these studies was expected, as the human and animal intestines serve as reservoirs (Jafari, Aslani & Bouzari 2012). Unlike most studies that focus on farms or slaughterhouses, the present study used a farm-to-fork approach, ensuring adequate evaluation of the presence and abundance of *E. coli* along the entire continuum. Thus, although *E. coli* was statistically significantly most abundant in the farm, substantial numbers were also recorded at the abattoir, whilst the lowest *E. coli* counts were found in the truck samples. The presence of *E. coli* at the abattoir revealed that although the scalding and singeing method has a significant impact in lowering the *E. coli* abundance in the carcass, it is still not enough to eliminate contamination during processing (Wu et al. 2009), especially during evisceration, as previously highlighted by Warriner et al. (2002). Whilst the abundance of *E. coli* in the faecal samples may not be considered a significant threat to human health, the considerable numbers in the meat portions could represent a meat safety challenge that needs to be addressed by ensuring that more efficient hygienic practices are applied to prevent packaging of contaminated meat. It has been demonstrated that meat protein is a potential source of bacterial foodborne disease because of its high protein content, water activity and approximately neutral pH, allowing the proliferation of bacteria (Jaja, Green & Muchenje 2018).

### Prevalence of diarrheagenic *E. coli* pathotypes

The DEC pathotypes continue to be a major health threat globally because of the disease outbreak-causing potentials. Thus, identifying all possible reservoirs of these pathotypes is essential to ensure public health. In the current study, the highest prevalence of DEC was found on the farm. This was logical because the samples isolated from the farm were predominantly faecal. Also, the number of samples isolated from the farm was much higher than those isolated from the truck and abattoir, increasing the chances of isolating the pathotypes at farm level. The prevalence of DEC in the truck after the loading of pigs was higher, indicating their possible faecal sources.

Although different DEC pathotypes, including EPEC, have been implicated in colibacillosis in pigs, ETEC is the most frequently isolated pathotype (García-Meniño et al. 2018). The ETEC *coli* is a major cause of severe diarrhea in suckling and weaning animals, and is a cause of significant losses in the African pig industry (Kagambega et al. 2012; Kylla et al. 2019). The relatively low prevalence of EPEC and ETEC could be because this study was conducted on healthy animals, and no incidence of diseased pigs was recorded throughout the study.

Apart from EPEC and ETEC, the other DEC pathotypes were also recorded in the current study, albeit at low prevalences (Table 1). This indicates that pigs constitute a significant reservoir of DEC pathotypes that could cause human infections (Borges et al. 2012; Kagambega et al. 2012). Interestingly, although EIEC is the most detected pathotype in the present study, this pathotype is rarely reported in pigs. Also, this pathotype was the only pathotype that was spread across all the sampling points in the continuum. Although this dominant occurrence could not be explained, it is important to note that this pathotype is highly implicated in bacillary dysentery in humans, particularly in low-income countries (Pasqua et al. 2017). It has also been implicated in foodborne disease outbreaks even in highly industrialised countries with improved hygiene and sanitation, such as the United States (Venkitanarayanan & Doyle 2003). Therefore, its detection across the continuum, including substantially at the abattoir, calls for stringent implementation of hygienic protocols in intensive pig farm systems. We also detected EHEC (1%), including O157:H7 (3%), at the farm level. Despite their low prevalence, this percentage was still significant because, although most pathogenic *E. coli* are infective at high doses, EHEC requires only ten cells to be infective (Limayem & Martin 2014).

### Antibiotic susceptibility profiles

The growing problem of antibiotic resistance has become a significant public health concern (Tanih et al. 2015). In South Africa, sulphonamides, tetracyclines, macrolides, ionophores, quinoxalines, glycolipids, polypeptides, streptogramins, oligosaccharides, phosphonic acid, nitroimidazoles and polymeric compounds are registered in various dosage for veterinary use and freely available to farmers for prophylaxis, metaphylaxis, growth promotion and infection treatment (Eagar & Naidoo 2017). This could explain the antibiotic resistance profiles observed, with most isolates showing resistance to tetracycline, chloramphenicol, ampicillin and trimethoprim-sulfamethoxazole (Figure 2a). The incidence of drug-resistant DEC pathotypes revealed that pork might pose a public health risk. Although tigecycline and imipenem recorded low resistance rates in the current study, they are still alarming because they are considered last-resort antibiotics for human use. The high percentage of MDR isolates obtained in the current study suggest that these organisms were exposed to high antibiotic use environments. This is substantiated by the fact that most of the MDR isolates recorded MARIs greater than 0.2. A MARI greater or equal to 0.2 indicated potential high-risk as the organisms probably originate from environments where antibiotics are extensively used (Teshome et al. 2020).

Diarrhea caused by foodborne pathogens such as DEC pathotypes is an important cause of death, especially in children in low- and middle-income countries (Jaja et al. 2018). The rise in MDR DEC aggravates the situation because MDR strains are known to limit treatment options. In order to minimise the risk and prevent microbial contamination along the food production chain, good hygiene manufacturing practice (GHMP) and hazard analysis critical control points (HACCP) practices should be implemented, in addition to appropriate retail and consumer meat handling and processing (Galli et al. 2016). In South Africa, meat safety control is a shared responsibility between the Department of Agriculture, Land Reform and Rural Development (DALRRD) and the Department of Health (DoH). Approaches to meat safety control include farm-to-fork control with the publication of HACCP regulations.

## Conclusion

The presence of MDR DEC pathotypes in animal food production environments and food of animal origin indicates that these animals serve as reservoirs and potential sources of these pathogenic organisms that could be transmitted to humans. This is especially in occupationally exposed workers and through consumption of undercooked pork. Adherence to good hygienic practices along the pig production continuum and thorough cooking are essential for mitigating the risk of transmission and infection and ensuring food safety.

## Appendix 1

**TABLE 1-A1:** Primers and controls used for pathotypes identification.

Target strain	Target gene	Primer Sequence (5´- 3´)	Positive control	References
EPEC/EHEC	*eae*	ATGCTTAGTGCTGGTTTAGG GCCTTCATCATTTCGCTTTC	DSM8695	Wang, G., Clark, C.G. & Rodgers, F.G., ‘*Escherichia coli* detection’, *Journal of Clinical Microbiology* 40(10), 3613–3619. https://doi.org/10.1128/JCM.40.10.3613-3619.2002
ETEC	*lt*	TCTCTATGTGCATACGGAGC CCATACTGATTGCCGCAAT	DSM10973	Omar, K.B. & Barnard, T.G., 2014, ‘Detection of diarrhoeagenic Escherichia coli in clinical and environmental water sources in South Africa using single-step 11-gene m-PCR’, *World Journal of Microbiology Biotechnology* 30, 2663–2671. https://doi.org/10.1007/s11274-014-1690-4
	*st*	TTTCCCCTCTTTTAGTCAGTCAACTG GGCAGGATTACAACAAAGTTCACA		
EAEC	*eagg*	AGACTCTGGCGAAAGACTGTATC ATGGCTGTCTAATAGATGAGAAC	DSM10974	
DAEC	*daaE*	GTCCGCCATCACATCAAAA TGGAACCCCGCTCGTAATATAC		
EIEC	*ipaH*	GTTCCTTGACCGCCTTTCCGATACCGTC GCCGGTCAGCCACCACCCTCTGAGAGTAC	DSM9025	Abia, A.L.K., Schaefer, L., Ubomba-Jaswa, E. & Le Roux, W., 2017, ‘Abundance of pathogenic Escherichia coli virulence-associated genes in well and borehole water used for domestic purposes in a peri-urban community of South Africa’, *International Journal of Environmental Research and Public Health* 14(3), 320. https://doi.org/10.3390/ijerph14030320
EHEC/0157H7	*stx*	GAGCGAAATAATTTATATGTG TGATGATGGCAATTCAGTAT	In-house O157:H7 strain	Ramirez Castillo, F.Y., Avelar González, F.J., Garneau, P., Marquez Diaz, F., Guerrero Barrera, A.L. & Harel, J., 2013, ‘Presence of multi-drug resistant pathogenic Escherichia coli in the San Pedro River located in the State of Aguascalientes, Mexico’, *Frontiers in Microbiology* 4, 147. https://doi.org/10.3389/fmicb.2013.00147
	*stx1*	CTGGATTTAATGTCGCATAGTG AGAACGCCCACTGAGATCATC		Abia, A.L.K., Schaefer, L., Ubomba-Jaswa, E. & Le Roux, W., 2017, ‘Abundance of pathogenic Escherichia coli virulence-associated genes in well and borehole water used for domestic purposes in a peri-urban community of South Africa’, *International Journal of Environmental Research and Public Health* 14(3), 320. https://doi.org/10.3390/ijerph14030320
	*stx2*	CCATGACAACGGACAGCAGTTCCT GTCAACTGAGCACTTTG		
	*flicH7*	TACCATCGCAAAAGCAACTCC GTCGGCAACGTTAGTGATACC		Ramirez Castillo, F.Y., Avelar González, F.J., Garneau, P., Marquez Diaz, F., Guerrero Barrera, A.L. & Harel, J., 2013, ‘Presence of multi-drug resistant pathogenic Escherichia coli in the San Pedro River located in the State of Aguascalientes, Mexico’, *Frontiers in Microbiology* 4, 147. https://doi.org/10.3389/fmicb.2013.00147

EPEC, enteropathogenic *E. coli*; EHEC, enterohemorrhagic *E. coli*; ETEC, enterotoxigenic *E. coli*; EAEC, enteroaggregative *E. coli*; DAEC, diffusely aggregative *E. coli*; EIEC, enteroinvasive *E. coli.*

**TABLE 2-A1:** Statistical difference between abundance of *E. coli* at the different sampling points.

Site	Average *E. coli* count, MPN/100 mL	Overall ANOVA *p*-value	Pair wise comparison (Tukey HSD *P*-value)	
FARM	1.008 × 10^6^	0.002**	Farm versus Truck	0.048*
TRUCK	0.0012 × 10^6^	-	Farm versus Abattoir	0.004**
ABATTOIR	0.0205 × 10^6^	-	Truck versus Abattoir	0.183

ANOVA, analysis of variance; HSD, honestly significant difference.

* , Statistical significance = *p* < 0.05;

** , Statistical significance = *p* < 0.01.

**TABLE 3-A1:** Prevalence of diarrheagenic *E. coli* along the pig production continuum.

Site	Number of DEC found	DEC found (%)	Overall Chi-square *p*-value	Pair wise comparison	
FARM	404	48.1	0.000***	Farm versus Truck	0.000***
TRUCK	7	16.2	-	Farm versus Abattoir	0.039*
ABATTOIR	63	39.1	-	Truck versus Abattoir	0.006**

DEC, diarrheagenic *E. coli.*

* , Statistical significance = *p* < 0.05;

** , Statistical significance = *p* < 0.01;

*** , Statistical significance = *p* < 0.001.

**TABLE 4-A1:** Multiple-antibiotic resistance index for all diarrheagenic *E. coli* pathotypes along the farm-to-fork continuum.

Bacterial ID	DEC pathotype	Source	MAR
B4(1)R3	DAEC	faecal	0
A5(6)R7	EPEC	faecal	0
W1(4)R8	EHEC	water	0
A1(2)R9	EPEC	faecal	0
TB1(2)	EIEC	truck	0
TA1(8)	EIEC	truck	0
CS1(10)	EIEC	abattoir	0
A1(6) R2	EPEC	faecal	0.05
A1(7)R2	EPEC	faecal	0.05
A1(8)R2	EPEC	faecal	0.05
A1(9)R2	EPEC	faecal	0.05
A1(10)R2	EPEC	faecal	0.05
B2(3)R2	EPEC	faecal	0.05
A1(3)R5	EAEC	faecal	0.05
A2(9)R5	DAEC	faecal	0.05
A4(2)R6	EIEC	faecal	0.05
A4(7)R6	EIEC	faecal	0.05
A1(5)R7	DAEC	faecal	0.05
A1(6)R7	EAEC	faecal	0.05
A1(8)R7	EIEC	faecal	0.05
A3(6)R7	EHEC	faecal	0.05
A3(10)R7	EHEC	faecal	0.05
B1(8)R7	DAEC	faecal	0.05
B1(10)R7	EHEC	faecal	0.05
B2(7)R7	EHEC	faecal	0.05
B3(6)R7	EHEC	faecal	0.05
A2(7)R8	EIEC	faecal	0.05
W1(2)R8	EIEC	water	0.05
W3(3)R8	EHEC	water	0.05
A4(3)R9	EIEC	faecal	0.05
A4(8)R9	EIEC	faecal	0.05
B4(4)R9	DAEC	faecal	0.05
B5(3)R9	EIEC	faecal	0.05
CAC1(1)	EIEC	abattoir	0.05
CAC1(2)	EIEC	abattoir	0.05
CAC2(7)	EIEC	abattoir	0.05
CAC3(10)	EIEC	abattoir	0.05
CR2(9)	EIEC	abattoir	0.05
CR2(10)	EIEC	abattoir	0.05
CS2(1)	EIEC	abattoir	0.05
CS2(3)	EIEC	abattoir	0.05
CS2(6)	EIEC	abattoir	0.05
CS4(5)	EIEC	abattoir	0.05
CS4(6)	EIEC	abattoir	0.05
B3(1)	EIEC	abattoir	0.05
H3(9)	EIEC	abattoir	0.05
H4(7)	EPEC	abattoir	0.05
A1(7)R1	EIEC	faecal	0.1
B1(2)R1	EIEC	faecal	0.1
B1(5)R1	EPEC	faecal	0.1
WA1(2)R1	EIEC	water	0.1
WB2(1)R1	EPEC	water	0.1
WB3(3)R1	DAEC	water	0.1
A1(3)R2	EPEC	faecal	0.1
A2(5)R2	EPEC	faecal	0.1
A2(7)R2	EPEC	faecal	0.1
B5(5)R2	EPEC	faecal	0.1
B5(4)R3	EIEC	faecal	0.1
A1(1)R4	ETEC	faecal	0.1
A1(5)R4	ETEC	faecal	0.1
A1(6)R4	ETEC	faecal	0.1
A1(8)R4	EIEC	faecal	0.1
A1(9)R4	EIEC	faecal	0.1
A1(10)R4	ETEC	faecal	0.1
A2(1)R4	EIEC	faecal	0.1
A2(2)R4	ETEC	faecal	0.1
A2(3)R4	EIEC	faecal	0.1
A2(4)R4	EIEC	faecal	0.1
A2(5)R4	ETEC	faecal	0.1
A2(6)R4	ETEC	faecal	0.1
A2(9)R4	DAEC	faecal	0.1
A4(1)R4	ETEC	faecal	0.1
A4(3)R4	ETEC	faecal	0.1
A4(5)R4	EIEC	faecal	0.1
A5(5)R4	EPEC	faecal	0.1
A5(6)R4	ETEC	faecal	0.1
B2(3)R4	ETEC	faecal	0.1
B3(1)R4	ETEC	faecal	0.1
A1(2)R5	EIEC	faecal	0.1
A1(5)R5	EIEC	faecal	0.1
A2(3)R5	EHEC	faecal	0.1
A4(9)R5	EIEC	faecal	0.1
A2(2)R6	EPEC	faecal	0.1
A2(7)R7	DAEC	faecal	0.1
A5(9)R7	ETEC	faecal	0.1
B1(2)R7	EAEC	faecal	0.1
B2(6)R7	ETEC	faecal	0.1
A1(5)R8	EIEC	faecal	0.1
B4(9)R8	EIEC	faecal	0.1
W1(1)R8	EIEC	water	0.1
A2(2)R9	EIEC	faecal	0.1
A3(7)R9	EIEC	faecal	0.1
A5(6)R9	EHEC	faecal	0.1
B1(1)R9	EIEC	faecal	0.1
B2(4)R9	EIEC	faecal	0.1
B2(6)R9	EPEC	faecal	0.1
TB1(1)	EIEC	truck	0.1
TB3(6)	EIEC	truck	0.1
CAC1(4)	EIEC	abattoir	0.1
CAC1(9)	EIEC	abattoir	0.1
CAC1(10)	EIEC	abattoir	0.1
CAC2(3)	EIEC	abattoir	0.1
CAC2(4)	EIEC	abattoir	0.1
CAC2(8)	EIEC	abattoir	0.1
CAC2(9)	EIEC	abattoir	0.1
CAC2(10)	EIEC	abattoir	0.1
CAC3(3)	EIEC	abattoir	0.1
CAC4(1)	EIEC	abattoir	0.1
CAC4(7)	EIEC	abattoir	0.1
CR1(9)	EIEC	abattoir	0.1
CR2(6)	EIEC	abattoir	0.1
CR2(7)	EIEC	abattoir	0.1
CS1(4)	EIEC	abattoir	0.1
CS1(8)	EIEC	abattoir	0.1
CS2(4)	EIEC	abattoir	0.1
CS2(7)	EIEC	abattoir	0.1
CS2(8)	EIEC	abattoir	0.1
CS2(9)	EIEC	abattoir	0.1
H2(8)	EIEC	abattoir	0.1
H3(7)	EIEC	abattoir	0.1
A1(9)R1	DAEC	faecal	0.15
A1(10)R1	EIEC	faecal	0.15
A2(8)R1	EIEC	faecal	0.15
B1(3)R1	EIEC	faecal	0.15
B1(7)R1	EPEC	faecal	0.15
B2(6)R1	EIEC	faecal	0.15
B3(9)R1	EIEC	faecal	0.15
A1(4) R2	EPEC	faecal	0.15
A2(3)R2	EPEC	faecal	0.15
A2(4)R2	EPEC	faecal	0.15
A2(8)R2	EPEC	faecal	0.15
A3(1)R2	EPEC	faecal	0.15
B2(1)R2	EPEC	faecal	0.15
B2(2)R2	EPEC	faecal	0.15
B2(6)R2	EHEC	faecal	0.15
B3(9)R2	EPEC	faecal	0.15
B4(6)R2	EPEC	faecal	0.15
A1(7)R3	EPEC	faecal	0.15
B1(2)R3	EPEC	faecal	0.15
B1(5)R3	EIEC	faecal	0.15
B1(7)R3	EIEC	faecal	0.15
A1(4)R4	EPEC	faecal	0.15
A2(7)R4	ETEC	faecal	0.15
A2(10)R4	ETEC	faecal	0.15
A3(10)R4	EIEC	faecal	0.15
A5(4)R4	ETEC	faecal	0.15
A5(7)R4	ETEC	faecal	0.15
A5(9)R4	ETEC	faecal	0.15
B3(4)R4	EPEC	faecal	0.15
A4(8)R5	EIEC	faecal	0.15
A2(6)R6	EPEC	faecal	0.15
A2(10)R6	EIEC	faecal	0.15
A5(2)R6	EPEC	faecal	0.15
A5(8)R6	EPEC	faecal	0.15
A5(9)R6	EPEC	faecal	0.15
A2(10)R7	EAEC	faecal	0.15
A3(8)R7	DAEC	faecal	0.15
A4(2)R7	EIEC	faecal	0.15
B2(3)R7	EPEC	faecal	0.15
B4(4)R7	EPEC	faecal	0.15
A1(2)R8	DAEC	faecal	0.15
A3(9)R8	EHEC	faecal	0.15
B2(6)R8	EHEC	faecal	0.15
W2(1)R8	EHEC	water	0.15
A1(5)R9	EPEC	faecal	0.15
A2(7)R9	EPEC	faecal	0.15
A4(9)R9	EPEC	faecal	0.15
B3(4)R9	EPEC	faecal	0.15
B4(2)R9	EIEC	faecal	0.15
TA2(9)	EIEC	truck	0.15
CAC1(8)	EIEC	abattoir	0.15
CR1(10)	EIEC	abattoir	0.15
CR2(3)	EIEC	abattoir	0.15
CS2(5)	EIEC	abattoir	0.15
CS4(3)	EIEC	abattoir	0.15
T3(10)	EIEC	abattoir	0.15
H3(10)	EIEC	abattoir	0.15
A2(7)R1	DAEC	faecal	0.2
A2(9)R1	DAEC	faecal	0.2
A3(7)R1	EIEC	faecal	0.2
A5(10)R1	EIEC	faecal	0.2
B5(9)R1	DAEC	faecal	0.2
B5(10)R1	DAEC	faecal	0.2
A1(2) R2	EPEC	faecal	0.2
A2(2)R2	DAEC	faecal	0.2
A5(6)R2	EPEC	faecal	0.2
B1(2)R2	ETEC	faecal	0.2
B1(7)R2	EPEC	faecal	0.2
B1(8)R2	EPEC	faecal	0.2
B1(9)R2	EPEC	faecal	0.2
B3(1)R2	EPEC	faecal	0.2
B3(2)R2	EPEC	faecal	0.2
B4(5)R2	EPEC	faecal	0.2
B5(6)R2	EPEC	faecal	0.2
B5(7)R2	EPEC	faecal	0.2
B5(9)R2	EPEC	faecal	0.2
WA2(3)R2	EPEC	water	0.2
WB1(1)R2	EHEC	water	0.2
A1(2)R4	ETEC	faecal	0.2
A1(3)R4	EIEC	faecal	0.2
A3(6)R4	ETEC	faecal	0.2
A3(9)R4	ETEC	faecal	0.2
B2(8)R4	ETEC	faecal	0.2
A3(3)R5	DAEC	faecal	0.2
A3(9)R6	EIEC	faecal	0.2
A2(9)R7	EHEC	faecal	0.2
A5(8)R7	EAEC	faecal	0.2
B1(9)R7	EHEC	faecal	0.2
B2(2)R7	EHEC	faecal	0.2
B3(3)R7	EHEC	faecal	0.2
B5(5)R7	EPEC	faecal	0.2
WA1(1)R7	EIEC	water	0.2
WA3(1)R7	EHEC	water	0.2
WB1(1)R7	EIEC	water	0.2
A4(2)R8	EHEC	faecal	0.2
B2(4)R8	ETEC	faecal	0.2
B2(9)R8	EHEC	faecal	0.2
B4(5)R8	EPEC	faecal	0.2
B5(2)R8	EPEC	faecal	0.2
B5(3)R8	EHEC	faecal	0.2
B5(8)R8	EAEC	faecal	0.2
A5(4)R9	ETEC	faecal	0.2
A5(10)R9	ETEC	faecal	0.2
B2(1)R9	EIEC	faecal	0.2
B3(2)R9	EIEC	faecal	0.2
B3(7)R9	EIEC	faecal	0.2
B4(7)R9	EIEC	faecal	0.2
TB1(7)	EIEC	truck	0.2
TA3(6)	EIEC	truck	0.2
B2(3)	EIEC	abattoir	0.2
A1(1)R1	ETEC	faecal	0.25
A2(2)R1	DAEC	faecal	0.25
A2(6)R1	EIEC	faecal	0.25
A3(3)R1	DAEC	faecal	0.25
A5(5)R1	EHEC	faecal	0.25
A5(8)R1	ETEC	faecal	0.25
B3(5)R1	ETEC	faecal	0.25
B4(1)R1	EPEC	faecal	0.25
WA1(3)R1	EPEC	water	0.25
WB1(1)R1	EPEC	water	0.25
WB1(2)R1	EPEC	water	0.25
WB3(2)R1	DAEC	water	0.25
A5(10)R2	EPEC	faecal	0.25
B1(3)R2	EPEC	faecal	0.25
B1(5)R2	EPEC	faecal	0.25
B2(7)R2	EPEC	faecal	0.25
B2(8)R2	EPEC	faecal	0.25
B2(9)R2	EPEC	faecal	0.25
B3(3)R2	EPEC	faecal	0.25
B4(8)R2	EPEC	faecal	0.25
B5(10)R2	EPEC	faecal	0.25
WA1(2)R2	EPEC	water	0.25
WA1(4)R2	EPEC	water	0.25
WA3(1)R2	EPEC	water	0.25
WA3(2)R2	EPEC	water	0.25
A2(8)R3	EPEC	faecal	0.25
A4(7)R3	EPEC	faecal	0.25
B4(3)R3	EIEC	faecal	0.25
B4(4)R3	EPEC	faecal	0.25
B4(10)R3	EIEC	faecal	0.25
A1(7)R4	ETEC	faecal	0.25
A5(1)R4	EPEC	faecal	0.25
A5(10)R4	ETEC	faecal	0.25
B2(5)R4	EIEC	faecal	0.25
B4(2)R7	EPEC	faecal	0.25
B4(3)R7	EPEC	faecal	0.25
B4(5)R7	EHEC	faecal	0.25
B2(1)R8	EPEC	faecal	0.25
B2(8)R8	EPEC	faecal	0.25
W3(2)R8	EIEC	water	0.25
A5(9)R9	DAEC	faecal	0.25
B3(3)R9	EPEC	faecal	0.25
CAC4(3)	EIEC	abattoir	0.25
CAC4(10)	EIEC	abattoir	0.25
CR1(3)	EIEC	abattoir	0.25
T3(9)	EIEC	abattoir	0.25
B3(5)	EIEC	abattoir	0.25
H2(3)	EIEC	abattoir	0.25
A1(8)R1	DAEC	faecal	0.3
A4(5)R1	EPEC	faecal	0.3
A4(7)R1	EPEC	faecal	0.3
A5(2)R1	DAEC	faecal	0.3
A5(4)R1	DAEC	faecal	0.3
A5(6)R1	DAEC	faecal	0.3
B3(8)R1	DAEC	faecal	0.3
B5(4)R1	EIEC	faecal	0.3
B5(7)R1	EAEC	faecal	0.3
A5(3)R2	EPEC	faecal	0.3
A5(4)R2	EPEC	faecal	0.3
A5(9)R2	EPEC	faecal	0.3
WA1(1)R2	EPEC	water	0.3
WB1(2)R2	EPEC	water	0.3
WB1(3)R2	EPEC	water	0.3
WB2(1)R2	EPEC	water	0.3
WB2(3)R2	EPEC	water	0.3
A4(3)R3	EPEC	faecal	0.3
A4(8)R3	EPEC	faecal	0.3
B2(8)R3	EPEC	faecal	0.3
B5(3)R3	EPEC	faecal	0.3
WA2(2)R3	EPEC	water	0.3
WB1(2)R3	EIEC	water	0.3
WB3(2)R3	EIEC	water	0.3
A4(4)R4	ETEC	faecal	0.3
A4(8)R4	ETEC	faecal	0.3
A4(10)R4	EHEC	faecal	0.3
B3(2)R4	ETEC	faecal	0.3
A3(9)R5	EIEC	faecal	0.3
W2(1)R5	EIEC	water	0.3
B3(9)R7	EPEC	faecal	0.3
B4(1)R7	EIEC	faecal	0.3
B5(3)R7	EIEC	faecal	0.3
B5(7)R7	EIEC	faecal	0.3
WA1(2)R7	EHEC	water	0.3
B1(4)R8	EHEC	faecal	0.3
B4(7)R8	EPEC	faecal	0.3
A3(6)R9	EIEC	faecal	0.3
CAC4(4)	EIEC	abattoir	0.3
CS1(6)	EIEC	abattoir	0.3
B2(5)	EIEC	abattoir	0.3
B3(8)	EIEC	abattoir	0.3
B3(9)	EIEC	abattoir	0.3
A5(1)R1	EIEC	faecal	0.35
B4(2)R1	EIEC	faecal	0.35
WA2(1)R1	DAEC	water	0.35
A1(1) R2	EPEC	faecal	0.35
A4(1)R2	EPEC	faecal	0.35
A5(8)R2	EHEC	faecal	0.35
B4(10)R2	EPEC	faecal	0.35
B5(4)R2	EAEC	faecal	0.35
WA2(2)R2	EPEC	water	0.35
WB2(2)R2	EPEC	water	0.35
A2(1)R3	EIEC	faecal	0.35
A5(5)R3	EHEC	faecal	0.35
B2(1)R3	EPEC	faecal	0.35
B3(9)R3	EPEC	faecal	0.35
A3(3)R4	ETEC	faecal	0.35
A3(4)R4	ETEC	faecal	0.35
A4(2)R4	EIEC	faecal	0.35
B2(1)R4	EIEC	faecal	0.35
W7R4	EIEC	water	0.35
B4(9)R7	EIEC	faecal	0.35
WA3(2)R7	ETEC	water	0.35
B4(6)R8	EPEC	faecal	0.35
A3(5)R9	EPEC	faecal	0.35
B1(4)R9	EPEC	faecal	0.35
CAC3(8)	EIEC	abattoir	0.35
T1(4)	EIEC	abattoir	0.35
T1(8)	EIEC	abattoir	0.35
H2(9)	EIEC	abattoir	0.35
A3(1)R1	DAEC	faecal	0.4
WA2(2)R1	EPEC	water	0.4
WA2(3)R1	ETEC	water	0.4
WA2(4)R1	DAEC	water	0.4
WA3(1)R1	EPEC	water	0.4
A3(3)R2	EPEC	faecal	0.4
A3(7)R2	EPEC	faecal	0.4
A3(8)R2	EPEC	faecal	0.4
A3(9)R2	EPEC	faecal	0.4
A3(10)	EPEC	faecal	0.4
A4(4)R2	EPEC	faecal	0.4
B4(4)R2	EPEC	faecal	0.4
A2(5)R3	EPEC	faecal	0.4
A2(10)R3	EIEC	faecal	0.4
A3(8)R3	EPEC	faecal	0.4
A3(9)R3	EPEC	faecal	0.4
A4(2)R3	EPEC	faecal	0.4
A4(9)R3	EPEC	faecal	0.4
A4(10)R3	EPEC	faecal	0.4
B4(8)R3	EIEC	faecal	0.4
B5(6)R3	EPEC	faecal	0.4
B5(10)R3	EIEC	faecal	0.4
A3(8)R4	EIEC	faecal	0.4
A4(6)R5	DAEC	faecal	0.4
A2(1)R8	EPEC	faecal	0.4
A3(1)R9	EHEC	faecal	0.4
A3(3)R9	EIEC	faecal	0.4
CAC3(7)	EIEC	abattoir	0.4
CR2(5)	EIEC	abattoir	0.4
A3(2)R2	EPEC	faecal	0.45
A3(5)R2	EPEC	faecal	0.45
B4(9)R2	EPEC	faecal	0.45
B5(3)R2	EPEC	faecal	0.45
A1(3)R3	EPEC	faecal	0.45
A1(5)R3	EPEC	faecal	0.45
A1(6)R3	EPEC	faecal	0.45
A1(9)R3	EIEC	faecal	0.45
A5(10)R3	EPEC	faecal	0.45
B3(2)R3	EPEC	faecal	0.45
WA2(3)R3	EPEC	water	0.45
WA3(3)R3	EIEC	water	0.45
WB1(4)R3	EPEC	water	0.45
W1(9)R5	EHEC	water	0.45
A3(2)R9	EIEC	faecal	0.45
T1(1)R12	EIEC	abattoir	0.45
B2(3)R1	EIEC	faecal	0.5
B2(5)R1	EIEC	faecal	0.5
A3(6)R2	EPEC	faecal	0.5
A4(8)R2	EPEC	faecal	0.5
A5(6)R3	EIEC	faecal	0.5
B1(6)R3	EIEC	faecal	0.5
B1(10)R3	EIEC	faecal	0.5
B5(9)R3	EIEC	faecal	0.5
WA1(2)R3	EPEC	water	0.5
WB3(1)R3	EIEC	water	0.5
A3(2)R4	EHEC	faecal	0.5
A5(3)R4	ETEC	faecal	0.5
A5(8)R4	EIEC	faecal	0.5
W1(1)R5	DAEC	water	0.5
W1(2)R5	EIEC	water	0.5
W1(3)R8	EIEC	water	0.5
B2(2)R1	DAEC	faecal	0.55
B3(3)R1	EAEC	faecal	0.55
A5(2)R2	EPEC	faecal	0.55
B4(2)R2	EPEC	faecal	0.55
WA3(3)R2	EPEC	water	0.55
B2(5)R3	EIEC	faecal	0.55
B1(5)R4	ETEC	faecal	0.55
W5R4	ETEC	water	0.55
A5(6)R5	EIEC	faecal	0.55
B1(1)R5	EIEC	faecal	0.55
W1(10)R5	DAEC	water	0.55
A2(3)R6	EIEC	faecal	0.55
A3(3)R6	EIEC	faecal	0.55
A5(7)R6	EHEC	faecal	0.55
A1(2)R7	EIEC	faecal	0.55
A1(6)R8	EIEC	faecal	0.55
A1(8)R8	EHEC	faecal	0.55
B4(7)R2	EPEC	faecal	0.6
A2(3)R3	EPEC	faecal	0.6
A3(3)R3	EPEC	faecal	0.6
B4(7)R3	EIEC	faecal	0.6
A3(7)R4	EIEC	faecal	0.6
B3(1)R5	EIEC	faecal	0.6
A1(4)R7	EIEC	faecal	0.6
B2(9)R7	DAEC	faecal	0.6
B3(1)R7	EIEC	faecal	0.6
A5(1)R2	EPEC	faecal	0.65
B2(7)R3	EPEC	faecal	0.65
B4(2)R4	EIEC	faecal	0.65
B4(3)R4	ETEC	faecal	0.65
W2(6)R5	EIEC	water	0.65
W2(8)R5	EIEC	water	0.65
W2(9)R5	EIEC	water	0.65
B4(6)R4	EIEC	faecal	0.7
B4(10)R4	EIEC	faecal	0.7
A3(1)R5	EIEC	faecal	0.7
B1(8)R5	EIEC	faecal	0.7
B2(6)R5	EIEC	faecal	0.7
B2(8)R5	EIEC	faecal	0.7
B4(2)R5	EIEC	faecal	0.7
B4(6)R5	EIEC	faecal	0.7
W2(10)R5	EIEC	water	0.7
A1(1)R7	EIEC	faecal	0.7
A1(3)R7	EIEC	faecal	0.7
W2(2)R5	EPEC	water	0.75
W2(3)R5	EIEC	water	0.75
B4(1)R2	EPEC	faecal	0.8
B1(9)R5	EIEC	faecal	0.8
B1(10)R5	EIEC	faecal	0.8
B2(3)R5	EPEC	faecal	0.8
B3(4)R5	EPEC	faecal	0.8
B4(1)R5	EIEC	faecal	0.8
B4(3)R5	EIEC	faecal	0.8
B4(4)R5	EIEC	faecal	0.8
B4(5)R5	EIEC	faecal	0.8
B4(7)R5	EIEC	faecal	0.8
B4(9)R5	EPEC	faecal	0.8
B4(10)R5	EIEC	faecal	0.8
B5(1)R5	EIEC	faecal	0.8
B5(2)R5	EIEC	faecal	0.8
B5(3)R5	EIEC	faecal	0.8
W1(7)R5	DAEC	water	0.8
B5(10)R5	EIEC	faecal	0.85
A5(2)R3	EHEC	faecal	0.9

DEC, diarrheagenic *E. coli*; MAR, multiple-antibiotic resistance; EPEC, enteropathogenic *E. coli*; EHEC, Enterohemorrhagic *E. coli*; ETEC, enterotoxigenic *E. coli*; EAEC, enteroaggregative *E. coli*; DAEC, diffusely aggregative *E. coli*; EIEC, enteroinvasive *E. coli.*
